# The spatial representations acquired in CA3 by self-organizing recurrent connections

**DOI:** 10.3389/fncel.2013.00112

**Published:** 2013-07-16

**Authors:** Erika Cerasti, Alessandro Treves

**Affiliations:** ^1^SISSA, Cognitive Neuroscience SectorTrieste, Italy; ^2^Collège de FranceParis, France; ^3^Kavli Institute for Systems Neuroscience and Centre for the Biology of Memory, NTNUTrondheim, Norway

**Keywords:** memory network, mossy fibers, associative plasticity, information theory, continuous attractors

## Abstract

Neural computation models have hypothesized that the dentate gyrus (DG) drives the storage in the CA3 network of new memories including, e.g., in rodents, spatial memories. Can recurrent CA3 connections self-organize, during storage, and form what have been called continuous attractors, or *charts*—so that they express spatial information later, when aside from a partial cue the information may not be available in the inputs? We use a simplified mathematical network model to contrast the properties of spatial representations self-organized through simulated Hebbian plasticity with those of charts pre-wired in the synaptic matrix, a control case closer to the ideal notion of continuous attractors. Both models form granular quasi-attractors, characterized by drift, which approach continuous ones only in the limit of an infinitely large network. The two models are comparable in terms of precision, but not of accuracy: with self-organized connections, the metric of space remains distorted, ill-adequate for accurate path integration, even when scaled up to the real hippocampus. While prolonged self-organization makes charts somewhat more informative about position in the environment, some positional information is surprisingly present also about environments never learned, borrowed, as it were, from unrelated charts. In contrast, context discrimination decreases with more learning, as different charts tend to collapse onto each other. These observations challenge the feasibility of the idealized CA3 continuous chart concept, and are consistent with a CA3 specialization for episodic memory rather than path integration.

## Introduction

It has been suggested on the basis of network models (McNaughton and Morris, 1987; Treves and Rolls, 1992) that new CA3 memory representations may be primarily established by inputs from the dentate gyrus (DG), while the input coming from the entorhinal cortex (EC) serves as cue for the retrieval process.

This hypothesis is consistent with behavioral evidence from spatial learning in rodents (McNaughton et al., 1989; Lassalle et al., 2000; Lee and Kesner, 2004) that indicates that dentate inactivation and dentate lesions disproportionately affect the acquisition of new spatial memories, rather than their retention. In a previous study we have formulated a mathematical model that allows assessing, in quantitative detail, the storage of spatial memories that could result from DG inputs (Cerasti and Treves, 2010). The model demonstrates that a sparse and entirely structure-less system of DG-CA3 projections can act effectively as a spatial random number generator, and impart considerable information content to distributions of CA3 place fields, by exploiting the very sparse activity seen across populations of granule cells (Chawla et al., 2005; Leutgeb et al., 2007). Intriguingly, much of this content has, however, a non-spatial character, due to the non-spatial and sparse nature of the connectivity. This non-spatial character is quantified in our study by the likelihood with which even distant locations in an environment can be confused with one another, when locations are decoded from the activity of a limited number of CA3 units.

Once the representation of a novel environment has been established in CA3, it is useful as a spatial memory if it allows the animal to reconstruct, from a partial cue, which environment it is in and/or its position within that environment (when both types of information are fully present in the inputs, there is no need for a memory system). An influential conceptual model (Samsonovich and McNaughton, 1997) posits that the CA3 recurrent network affords these abilities by using the DG-driven representations as continuous attractors. Among the *N* dimensions of its activity space, a continuous attractor based on *N* units spans a “chart” of only 1, or 2, or 3 dimensions, which correspond to those of physical space, e.g., 2 in the case of a planar environment. Along the other *N*-2 dimensions activity is attracted toward the chart, corresponding to the recognition of the environment, whereas activity can slide effortlessly along the 2 dimensions of the chart, guided e.g., by path integration, maintaining a memory of the current position. Learning arbitrary novel environments implies an ability to keep multiple charts in memory, in the same network.

Such a conceptual model is clear in principle, but is it realizable in practice? It is not yet understood, despite early studies (Samsonovich and McNaughton, 1997; Battaglia and Treves, 1998), how many multiple charts could be established and maintained by unsupervised synaptic plasticity on the recurrent CA3 connections. Even less, how close they would be to continuous attractors. Here, we use a simplified network model to show that what can be created by self-organization is considerably distant from a continuous attractor, and closer to a bundle of loosely organized discrete point-like attractors.

## Methods

### Basic model

The model we consider is an extended version of the one used in our previous study (Cerasti and Treves, 2010), where the firing rate of a CA3 pyramidal cell, η_*i*_, was determined, as the one informative component, by the firing rates {β} of DG granule cells, which feed into it through mossy fiber (MF) connections. The model used for the neuron was a simple threshold-linear unit (Treves, 1990), so that the firing of the unit is produced by an activating current (which includes several non-informative components) which is compared to a threshold:

(1) $$\eta_{i}(\vec{x})=g{\left[\sum_{j}c_{ij}^{\text{MF}}J_{ij}^{\text{MF}}\beta_{j}(\vec{x})+{\tilde{\delta }}_{i}-\tilde{T}\right]}^{+}$$

where *g* is a gain factor, while [·]^+^ equals the sum inside the brackets if positive in value, and zero if negative. The effect of the current threshold for activating a cell, along with the effect of inhibition, and other non-informative components, are summarized into a single subtractive term, with a mean value across CA3 cells expressed as $\tilde{T}$, and a deviation from the mean for each particular cell *i* as ${\tilde{\delta }}_{i}$, which acts as a sort of noise; threshold and inhibition, in fact, while influencing the mean activity of the network, are supposed to have a minor influence on the coding properties of the system. In the earlier reduced model, however, $\tilde{T}$ and ${\tilde{\delta }}_{i}$ also included the effect of other cells in CA3, through recurrent collateral (RC) connections, and that of the perforant path (the input coming from the EC), both regarded as unspecific inputs—this based on the assumption that information is driven into a new CA3 representation solely by MF inputs. In this study, instead, since we are interested in the ability of the RC system to retrieve and express spatial representations, we separate out the RC contribution, and redefine $\tilde{T}$ and ${\tilde{\delta }}_{i}$ into *T* and δ_*i*_ —which sum the remaining unspecific inputs, including the perforant path, not analyzed here:

(2) $$\eta_{i}(\vec{x})=g{\left[\sum_{j}c_{ij}^{\text{MF}}J_{ij}^{\text{MF}}\beta_{j}(\vec{x})+\sum_{k}c_{ik}^{\text{RC}}J_{ik}^{\text{RC}}\eta_{k}(\vec{x})+\delta_{i}-T\right]}^{+}$$

Connections between cells are indicated by the fixed binary matrices {*c*^MF^}, {*c*^RC^}, whose non-zero elements (which take value 1) represent the existence of anatomical synapses between two cells. The synaptic efficacies are instead indicated by the matrices of weights {*J*^MF^}, {*J*^RC^}, whose elements are allowed to take positive values. The notation is chosen to minimize differences with our previous analysis of other components of the hippocampal system (e.g., Treves, 1990; Kropff and Treves, 2008).

The firing rates of the various populations are all assumed to depend on the spatial position $\vec{x}$ of the animal; the time scale considered for evaluating the firing rate is of order the theta period, about a hundred msec, so the finer temporal dynamics over shorter time scales is neglected. Note that time is not explicitly included in the above equations, which only describe the end result of the dynamics at the theta scale. To be precise, in the simulations, we take a time step to correspond to 125 ms of real time, or a theta period. We assume in fact that the simulated rat moves at a speed of 20 cm/s, so during one step the rat moves by 2.5 cm. In support of the plausibility of this choice, recent evidence indicates that within each theta period only one spatial representation tends to be dynamically selected (Jezek et al., 2011).

### The storage of new representations

The important novel ingredient that was introduced by Cerasti and Treves (2010), and that makes the difference from previous models of self-organizing recurrent networks, is a realistic description of the patterns of firing in the inputs, i.e., in the DG. As the virtual rat explores the new environment, the activity $\beta_{j}(\vec{x})$ of DG unit *j* is determined by the position $\vec{x}$ of the animal, according to the expression:

(3) $$\beta_{j}(\vec{x})=\sum_{k=0}^{Q_{j}}\beta_{0}e^{-{\left(\vec{x}-{\vec{x}}_{jk}\right)}^{2}/2\sigma_{f}^{2}}$$

The firing rate of the granule cells is then a combination of *Q*_*j*_ Gaussian functions, resulting in “bumps,” or fields in the firing map of the environment, centered at random points ${\vec{x}}_{jk}$. The environment is taken to have size *A*, and the fields are defined as all having the same effective size π(σ_*f*_)^2^ and height β_0_. *Q*_*j*_, which indicates the multiplicity of fields of DG cell *j*, is drawn from a Poisson distribution:

(4) $$P\left(Q_{j}\right)=\frac{q^{Q_{j}}}{Q_{j}!}e^{-q}$$

with mean value *q* (and we take *q* = 1.7), which roughly fits the data reported by Leutgeb et al. (2007). According to the same experimental data, we assume that only a randomly selected fraction *p*_*DG*_ << 1 (here set at *p*_*DG*_ = 0.033) of the granule cells are active in a given environment. Hence population activity is sparse, but the firing map of individual active granule units need not be sparse (it would only be sparse if *q* π(σ_*f*_)^2^/*A* << 1, which we do not assume to be always the case).

The activity of DG units determines the probability distribution for the firing rate of any given CA3 pyramidal unit, once the connectivity level between the two layers has been fixed: $\left\{C_{ij}^{\text{MF}}\right\}=0,\;1$ with $P\left(C_{ij}^{\text{MF}}=1\right)=\frac{C^{\text{MF}}}{N_{\text{DG}}}\equiv c^{\text{MF}}$. In agreement with experimental data, we set *C*^MF^ = 50, a value in the range of the ones providing an optimal information transmission from DG to CA3 (Cerasti and Treves, 2010). The MF synaptic weights are set to be uniform in value, *J*^MF^_*ij*_ ≡ *J*, and similarly *J*^RC^_*ij*_ ≡ *J*^RC^_0_ initially. Subsequently, during the learning phase, RC weights are modified according to the simulated learning process and under the influence of the input coming from the MF connections. Following the simplified hypothesis that the MFs carry all the information to be stored without contributing anything to the retrieval process, which is left to the RC, MF weights are kept fixed to their initial values *J*; note that we have found, in our earlier study, that MF connections appear to be inadequate, even when associatively plastic, to support retrieval of spatial representation (Cerasti and Treves, 2010).

The connectivity among CA3 cells is given by the matrix $\left\{C_{ij}^{\text{RC}}\right\}=0,\;1$ with $P\left(C_{ij}^{\text{RC}}=1\right)=\frac{C^{\text{RC}}}{N_{\text{CA3}}}\equiv c^{\text{RC}}$, where *C*^RC^ = 900 in most simulations. The activity of the network is regulated by the constraint we impose on its mean and on its sparsity *a*_CA3_, i.e., the fraction of the CA3 units firing significantly at each position, which is an important parameter affecting memory retrieval (Treves, 1990; more precisely, *a*_CA3_ = 〈η_*i*_〉^2^/〈η^2^_*i*_〉). Here we set the sparsity of each representations as *a*_CA3_ = 0.1, in broad agreement with experimental data (Papp et al., 2007), and at each time step we regulate the threshold *T* accordingly, to fulfill such requirement, while keeping the mean activity 〈η_*i*_〉 = 0.1 by adjusting the gain *g*.

### Recurrent collateral plasticity

During the learning phase, the activity of CA3 is driven by DG inputs, and RC connections contribute through weights uniformly set to their initial value *J*^RC^_0_. While the virtual rat explores the environment, RC weights are allowed to change according to an associative “Hebbian” learning rule, such that the total change in the synaptic weights is given as a sum of independent terms:

(5) $$\Delta J_{ij}^{RC}(t)=\gamma \eta_{i}(t)\left(\eta_{j}(t)-\Lambda_{j}(t)\right)$$

where Δ*J*_*ij*_(*t*) indicates the variation of the connection weight between cells *i* and *j* occurring at a given time step t, η_*i*_, and η_*j*_ are the postsynaptic and presynaptic firing rate, while γ is the learning rate. This associative learning rule includes the contribution of a trace, Λ, of the recent past activity of the presynaptic cell, defined as:

(6) $$\Lambda_{j}(t)=\frac{1}{\tau }\sum_{t_{s}=1}^{\tau }\eta_{j}(t-t_{s})$$

where τ is taken equal to 14 time steps (1750 ms). RC weights are forced to be non-negative, so they are reset to zero each time they become negative. Moreover, the total of the synaptic weights afferent to a single postsynaptic CA3 cell is normalized at the end of the learning process, so that $\sum_{j=1}^{C^{\text{RC}}}J_{ij}^{\text{RC}}=1$ per each CA3 cell. In words, the synaptic plasticity on recurrent connections allows the system to store the information about the current environment conveyed by MF inputs; such information is expressed in the form of place-like patterns of activity in CA3 units, and the Hebb-like learning rule strengthens the connections between units that show overlapping fields.

### Pre-wired exponential connectivity model

In contrast to and as a control for this self-organizing connectivity, we also consider the case of a model network endowed with a pre-wired connectivity. The structure of connections is functional to the establishment of a quasi-continuous attractor surface, expressed by synaptic weights that follow an exponential decreasing function of the distance between place field centers (Tsodyks and Sejnowski, 1995; Samsonovich and McNaughton, 1997; Battaglia and Treves, 1998; Roudi and Treves, 2008). The weight of the connection between cell *i* and *j* is then written:

(7) $$J_{ij}^{\text{RC}}=e^{-\left|{\vec{x}}_{i}^{c}-{\vec{x}}_{j}^{c}\right|/\lambda }$$

if the two cells *i* and *j* both have place fields in the given environment with centers ${\vec{x}}_{i}^{c}$ and ${\vec{x}}_{j}^{c}$ (the largest field is chosen for each cell that has more than one), while *J*_*ij*_ = 0 if at least one of them has no place fields. We usually set here the characteristic spread of the connectivity to λ = 5 cm, and to λ = 10 cm in some control simulations, and the size of the environment is taken to be A = 1 × 1 m; see below. The synaptic efficacies, in this control model, result from an artificial construction based on a precise and quite narrow exponential function; however, the distribution of place fields at the basis of such construction stems from the DG input, so that the place fields of CA3 units are randomly distributed in space, and not regularly arranged (Cerasti and Treves, 2010).

### Simulations

The mathematical model described above was simulated with a network of typically 45000 DG units and 1500 CA3 units (although in the “quick” multi-chart simulations described below the number of DG units goes down to 15,000 and that of CA3 units to 500). A virtual rat explores a continuous two dimensional space, intended to represent a 1 sqm square environment but realized as a torus, with periodic boundary conditions. For the numerical estimation of mutual information, the environment is discretized in a grid of 20 × 20 locations, whereas trajectories are in continuous space, but in discretized time steps. In each time step (of 125 ms) the virtual rat moves 2.5 cm in a direction similar to the direction of the previous time step, with a small amount of noise. To allow construction of a full localization matrix with good statistics, simulations are run for typically 400,000 time steps (nearly 35 h of virtual rat time). Given the choice of periodic boundary conditions, made to avoid border effects, the longest possible distance between any two sampling locations is equal to 14.1 grid units, or 70 cm.

### Scaling up the network

Different network sizes as used in Figure 2 (where the number of units in the CA3 layer is indicated) are implemented in the simulations through the multiplication of the following parameters by a given factor: the total number of DG units, *N*^tot^_DG_, the total number of CA3 units, *N*^tot^_CA3_, the number of RC connections, *C*^RC^. The multiplicative factors, relative to the reference model with 1500 CA3 units, are 1/3, 1, 5/3, 7/3, 10/3, 16/3. The connectivity between DG and CA3 stays unchanged, with always 50 MFs projecting to a single CA3 cell.

### Threshold setting in CA3

CA3 units fire according to Equation (2), with the threshold *T* hypothesized to serve to adjust the sparsity *a*_CA3_ of CA3 activity to its required value. The sparsity is defined as:

(8) $$a_{CA3}={\left(\sum \eta_{i}\left(\vec{x}\right)\right)}^{2}\sum \eta_{i}^{2}\left(\vec{x}\right)$$

and it is set to *a*_*CA*3_ = 0.1. This implies that the activity of the CA3 cell population is under tight inhibitory control.

### Hebbian learning process

Before and during the learning session, all recurrent connections weights take the same value *J*^RC^_0_; after the learning phase, they take the values resulting from the sum of all modifications occurred during the session, and described by Equation (5), with learning rate γ. The trajectory of the virtual rat during the learning session is a random path, extended over a time long enough for it to effectively visit repeatedly all possible locations in space: 10,000 time steps to cover 400 locations (in each time step the rat is made to move half a grid unit). This is taken to correspond to about 20 min of exploration in real time. Such synaptic modifications start to have an effect on the CA3 firing rate only at the end of the learning session, when the RC weights are updated to their new values. In the “quick” multi-chart simulations below, the learning rate was sped up by a factor 20, γ = 0.002: in those simulations, each learning session (per each learned environment, or chart) lasted 3000 time steps, with the higher learning rate intended to partially compensate for the fewer time steps.

### Turning off the DG input

For simulations aimed at describing attractor properties, in each position of the virtual rat activity is allowed to reverberate for 15 iterations; with a full DG input during the first one, an input reduced to 1/3 during the second, and to 0 for the remaining 13 time steps. The final position is then inferred by comparing the population vector in the last iteration, i.e., the vector with components [η_1_(*t*), …, η_*i*_, (*t*) …, η_*N*_(*t*)] with the set of all templates, which are the population vectors corresponding to 400 possible locations in space (on a the 20 × 20 grid), measured when the DG input is on. The position relative to the template that is correlated the most with the actual population vector is taken to be the final position resulting from attractor dynamics elicited by the initial spatial input. Most correlated means having the smallest Euclidean vector distance. For simulations aimed at assessing the storage of multiple environments, for each position of the virtual rat the activity reverberates for only 5 iterations, to save CPU time; with the full DG input during the first 2, reduced to 1/3 on the third, and to 0 for the remaining 2 iterations.

Note that in order to assess the information content stored in the network, in this study we always probe the network by first presenting it with a full cue. Of course, the real memory capabilities of the CA3 network should be appreciated also by providing it with partial cues, possibly conveyed to the system by the direct PP input from EC, so that information is actually retrieved from the network and not merely relayed onwards. This can be the subject of future studies.

### Decoding procedure and information extraction

At each time step, the firing vector of a sample of CA3 units is compared to each of the “template” vectors recorded, in previous test trials, at each position in the 20 × 20 grid, for the same sample of units. The position of the closest template, in the Euclidean distance sense, is taken to be the decoded position at that time step, for that sample. At the last iteration, this is taken to be the “final” position, reached through network dynamics, when the virtual rat is in a fixed position, corresponding to the “initial” one conveyed to the CA3 network by the MF inputs, as illustrated also in Figure 2. This procedure has been termed maximum likelihood Euclidean distance decoding (Rolls et al., 1997). The frequency of each pair of decoded and real positions are compiled in a so-called “confusion matrix,” or localization matrix, that reflects the ensemble of conditional probabilities $\left\{P(\left\{\eta_{i}\right\}\;|\vec{x})\right\}$ for that set of units. All information measures from simulations are obtained constructing the full localization matrix $Q(\vec{x},\;{\vec{x}}^{\prime })$ (Cerasti and Treves, 2010). If the square environment is discretized into 20 × 20 spatial bins, this is a large 400 × 400 matrix, which requires of order 160,000 decoding events to be effectively sampled. We run simulations with trajectories of 400,000 steps. In all the information measures we report, we have also corrected for the limited sampling bias, as discussed by Treves and Panzeri (1995). In our case of spatial information, the bias is essentially determined by the spatial binning we used (20 × 20) and by the decoding method (Panzeri et al., 1999). Should decoding “work” in a perfect manner, in the sense of always detecting the correct position in space of the virtual rat, the confusion matrix would be the identity matrix. From the confusion matrix obtained at the end of the simulation, the amount of information is extracted, and plotted versus the number of CA3 units present in the sample. We average extensively over CA3 samples, as there are large fluctuations from sample to sample, i.e., for each given number of CA3 units we randomly pick several different groups of CA3 units and then average the mutual information values obtained.

The decoding and information extraction procedure is used similarly for the evaluation of context discrimination: at each time step a vector of a sample of CA3 units is compared to each of four “template” vectors recorded previously in four different environments at the same location, for the same sample of units. The environment corresponding to the closest template, in the Euclidean distance sense, is taken to be the decoded environment at that time step, for that sample.

### Fitting

We fit the information curves obtained in simulations to exponentially saturating curves as a function of *N*, in order to get the values of the two most relevant parameters that describe their shape: the initial slope *I*_1_ (i.e., the average information conveyed by the activity of individual units) and the total amount of information *I*_8_ (i.e., the asymptotic saturation value). The function we used for the fit is:

(9) $$I(N)=I_{\infty }\left(1-e^{-NI_{1}I_{\infty }}\right)$$

In most cases the fit was in excellent agreement with individual data points, as expected on the basis of previous analyses (Samengo and Treves, 2000).

## Results

We simulate a network of CA3 units, receiving input from DG units with multiple, disorderly arranged place fields. Recurrent connections are inserted and contribute to network dynamics either through a “self-organized” weight distribution resulting from an associative learning process or, as a control, through a “pre-wired” distribution manually set by using an exponential function of the place field distance, between pairs of CA3 units with a place field (in some analyses, we shall consider also, as further controls, recurrent weights fixed at their uniform initial value, or reshuffled one with the other). The dynamics is analyzed when the input coming from the DG units is either on, to characterize externally driven representations, or turned off after probing the network with a full cue, to characterize instead memory-driven “attractors.” The noise level is kept very low (δ = 0.002) in order to probe the microstructure of the spatial attractors, and is then raised (δ = 0.1) to test their robustness. Parameters for the reference network are listed in Table 1.

**Table 1:** **Parameters**.

**Parameter**	**Symbol**	**Standard value**
Number of DG units in the network	*N*^tot^_DG_	45000
Number of CA3 units in the network	*N*^tot^_CA3_	1500
Probability a DG unit is active in one environment	*p*_DG_	0.033
Number of DG inputs to a CA3 unit	*C*^MF^	50
Number of RC inputs to a CA3 unit	*C*^RC^	0.6 × N^tot^_CA3_
Mean number of fields per active DG unit	*q*	1.7
Mean number of fields activating a CA3 unit	μ	*C*^MF^ × *p*_DG_ × *q* = 2.833
Strength of MF inputs	*J*	1 (or 2.833/μ)
Initial strength of RC inputs	*J*^RC^_0_	1/*C*^MF^
Noise affecting CA3 activity	δ	0.002 (in units in which β_0_ = 2.02)
Sparsity of CA3 activity	*a*_CA3_	0.1
Learning rate	γ	0.0001
Temporal trace window	τ	14 time steps (or 1750 ms)

*Values used in the standard version of the model with recurrent collaterals*.

### Self-organized 2D attractors are reasonably precise, but not accurate

**Figure 1:**
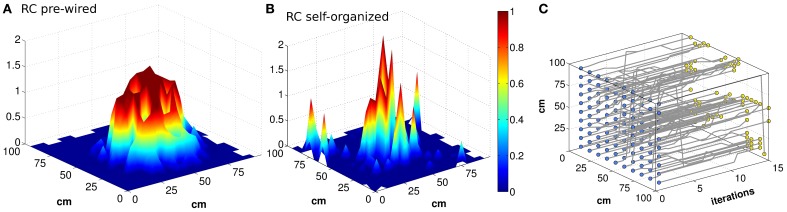
**Bumps of activity are rough, and tend to drift**. Activity packets showing the localization of the firing pattern, when the virtual rat is in the position of coordinates (*x*,*y*) = (55,45) (**A** and **B**). The firing rates of all the units active in the given environment, among *N_CA3_* = 1500 units, are plotted in the position of their (main) place field centers, when noise is very low (δ = 0.002). The attractor bump in a network with connectivity pre-wired following an exponential function **(A)** is much smoother than in a network with connectivity resulting from a Hebbian self-organizing process **(B)**, both expressed when the DG input is turned off. The trajectory of the activity bump over 16 iterations is outlined for each initial position **(C)** (the iterations are taken to be included within one 125 ms period during theta). The example in **(C)** is with pre-wired exponential connectivity.

**Figure 2:**
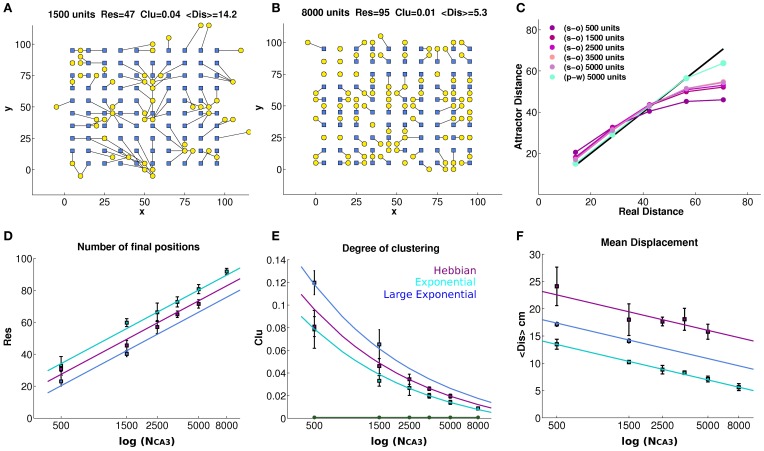
**Finite size effects eventually vanish, but the tendency to drift persists**. The bump tends to drift from any initial position in the environment, encoded in the inputs, to the final position decoded from the complete CA3 activity vector in the self-sustaining attractor (**A** and **B**), even when noise is very low (δ = 0.002). Each initial position (blue square) is connected to its final position (yellow circle) through a black line, showing strong drift of the bump as a result of turning off DG inputs, in a network of 1500 units with connectivity self-organized through Hebbian learning **(A)**, and much weaker drift in a network of 8000 units with pre-wired exponential connectivity and spread λ = 1 **(B)**. The panels **(A)** and **(B)** reports the number of distinct final positions *Res*, the degree of clustering *Clu* and the mean displacement from initial to final position < *Dis* >, whose average values over simulations with different network sizes are reported in the bottom row. **(C)** The average distance between final positions corresponding to a given input distance along the diagonal of the torus. As the network gets larger the global metric distortion of a self-organized net is reduced, approaching the black (identity) line, but very slowly. In contrast, a pre-wired network is much less distorted (light blue). **(D)** The mean number of final locations, obtained by providing 100 input locations, is plotted versus the size of the network (number of units in the CA3 layer) for networks with exponential pre-wired and with self-organized weights, when noise is very low (δ = 0.002). The y-axis indicates the number of different final positions, after the removal of the input. Values are averaged over 4 different seeds, and standard errors of the mean are indicated. The cyan data points indicate the values obtained from a network with exponential weights, with the standard spatial decay constant equal to 1 grid unit (or 2 grid units for the blue data points) while the purple points indicate values obtained from a network with Hebbian learning. Data points are seen to approximate the logarithmic trends explained in the text. **(E)** Degree of clustering of the final positions. A measure of clustering is plotted as a function of network size, for networks with exponential pre-wired weights and with Hebbian learning (same color coding **D** as). The new green line indicates the same *Clu* measure for the initial locations that, by construction, are regularly arranged on a grid 10 × 10. **(F)** The mean displacement, following the removal of the input, between the initial position and the final position.

Can the recurrent network in CA3 maintain a precise memory of the position in space of the animal, over realistic time scales, in the absence of afferent inputs? Neural activity in our CA3 model does indeed maintain a localized character, for quite some time after DG inputs are turned off, as illustrated by the examples in Figures 1A,B. The figure, however, indicates that the spatial code expressed by the model is rather far from the idealized notion of a smooth continuous attractor, which can sustain a distinct pattern of activity as a bell-shaped bump in each location of the environment. The control case of a pre-wired, finely tuned exponential connectivity, with DG inputs turned off, seems reasonably smooth (Figure 1A), while the self-organized bump in Figure 1B appears rather noisy. Bumps are in fact equally noisy also when the DG inputs are still on (not shown). Further, the example in Figure 1C indicates that the position of the bump drifts considerably when turning off the input. The idealized notion can only be crudely approximated with a network of finite size (Tsodyks and Sejnowski, 1995; Hamaguchi and Hatchett, 2006; Papp et al., 2007; Roudi and Treves, 2008). Are the irregularity and the drift merely finite size effects, due to the limited size of our simulations, which disappear for networks of real-brain size? Figure 2 shows that the imprecision and the tendency to drift are reduced when the network is larger. To quantify the exact extent to which recurrent network dynamics approach the ideal notion of a continuous attractor, we study how the effective spatial resolution and spatial memory accuracy of the code change, when increasing in discrete steps the number of units in both the DG and the CA3 population, and the RC connectivity. Different network sizes are indicated in Figure 2 by the number of units in the CA3 layer (see Methods).

Figure 2D quantifies spatial resolution by showing the number *Res* of different final positions obtained when the input is turned off, starting the network in 100 distinct initial positions. We can see that, already with 8000 units (Figures 2B,D), the spatial resolution of the pre-wired system can be quite high, failing to represent only about 5% of the initial locations, in the sense that the network reaches 95 distinct final positions. Note that the 100 initial positions are always taken as the positions at the vertices of a 10 × 10 grid (Figures 2A,B), embedded in the simulated 100 × 100 cm environment at every fourth node of the finer 20 × 20 grid used to produce population vector templates for decoding; and note that even distinct final positions do not, in general, coincide with the initial ones. As shown in Figure 2D, the spatial resolution of the attractor increases as the population of model CA3 neurons gets larger, both with pre-wired and with self-organized connectivity. This increase is intuitive; in fact with about a third of the CA3 cells active in the given environment, as a result of the DG input (Cerasti and Treves, 2010), an increase in population size implies an increase in the number of CA3 place fields randomly distributed over the environment. As a consequence, the firing level of units in the activity packet, determined by RC weights, would approximate better a smooth decrease with distance from the center (necessary for a truly continuous attractor, Tsodyks and Sejnowski, 1995) if a larger number of fields is available. In the case of the smallest network in Figure 2D, 500 units, indeed the poor resolution is due to the fact that only around 150 place field are present to cover the environment. The network with pre-wired and finely tuned (λ = 1) connectivity appears to have only marginally better resolution, across sizes, than the self-organized one, which shows in turn slightly better resolution than the pre-wired but more coarsely tuned network (λ = 2). The Figure 2D also shows, as guides to the eye, the straight lines which on the semi-logarithmic scale of the graph correspond to the trends:

(10) $$\text{Res}=-90+46\log_{10}\left(\frac{N_{CAs}}{\lambda }\right)$$

where the *Res* data-points for the self-organized network are reasonably well fit by taking an effective λ = 1.4. It is not clear at the stage whether these lines are just guides to the eye, or whether they really describe the scaling of finite size effects; this issue is left for future analysis. What they indicate is the size of the network for which *Res*~100, that is, how large the CA3 network has to be, not to display the imprecision expressed by the collapsing together of the bumps of activity originated on a 10 × 10 grid of initial positions. We can extrapolate *N*_*CA*3_ ~13,500 for the pre-wired network with finely tuned connectivity (λ = 1); *N*_*CA*3_ ~27,000 with coarser connectivity (λ = 2); and *N*_*CA*3_ ~18,900 with self-organized connectivity. Although approximately derived, such values are well below the size of the CA3 network in the rat, suggesting that lack of precision in representing distinct position may be a minor concern in the real rat brain.

To further test this hypothesis, we can also quantify fluctuations in the density of final positions, that is, the degree of clustering in their distribution, *Clu*, by calculating a weighted average for each pair *k, l* of final positions, and for each network realization, as follows:

(11) $$\text{Clu}=\frac{1}{S(S-1)}\sum_{k\neq l}^{S(S-1)}e^{-{\left({\vec{x}}_{k}-{\vec{x}}_{i}\right)}^{2}}$$

where *S* is the total number of locations, 100 in our case. The *Clu* measure takes its minimum (optimal) value *Clu*~0.00075 when the final positions are uniformly distributed in the environment, and its maximal (worst) value *Clu* = 1 when they are all clustered together. The values taken in our CA3 simulations are shown in Figure 2E, where the *Clu* value for the initial positions (the minimum one) is also indicated as a reference. The degree of clustering is much higher than the minimum reached for initial positions, indicating that several pairs of final positions do collapse onto each other or nearly so, yielding large contributions to the sum in Equation (6). The *Clu* measure thus quantifies the impression that final locations are not well distributed, and that the quasi-continuous attractor is locally distorted and “wrinkled.” One sees that the distortion is progressively reduced, however, with increasing network size. Again, one can approximately fit to the data-points curves, which in this case describe the trends:

(12) $$Clu=-0.016+2.12\sqrt{\lambda /N_{CA3}}$$

where for the self-organized network we can again take an effective value λ = 1.4. Again, we see that the pre-wired and finely tuned network leads to an attractor only marginally smoother than the self-organized one, which in turn is smoother than the more coarsely tuned pre-wired network. Extrapolating the curves to the optimal value *Clu*~0.00075, we can extract approximate network sizes beyond which clustering effects disappear. They turn out to be *N*_*CA*3_ ~16,000 for the pre-wired network with finely tuned connectivity (λ = 1); *N*_*CA*3_ ~32,000 with coarser connectivity (λ = 2); and *N*_*CA*3_ ~22,500 with self-organized connectivity. These values are similar, although somewhat larger, than those extrapolated from the resolution measures, again suggesting that for a real life rodent hippocampus spatial precision is not a major limitation. The network pre-wired with more coarsely tuned connections extending over double the distance (λ = 2) is significantly poorer, both in spatial resolution and in the degree of undesirable clustering, than the self-organized network (Figures 2D,E), indicating that the latter self-organizes rather precisely, and that the superiority of the control, exponentially pre-wired connections only arises due to their very finely tuned spatial coding.

Both the resolution and clustering measures of Figures 2D,E indicate a somewhat granular, but *locally* precise spatial code, able to discriminate neighboring position to a reasonable degree. They do not, however, tell us much about the *global* metric of the attractor surface, that is, whether the distance between any two real positions is reflected in the distance of the corresponding attractor representations. Such metric is distorted by bump drift. To graphically visualize what happens to a bump, we can look at Figure 1C; for each initial position, it is possible to follow the drift of the bump resulting from the turning off of the MF input and we can get an idea of the distortion the quasi-continuous attractor undergoes. This is expressed in Figure 2F, where the mean distance <*Dis*> is shown between initial positions and positions reached after 10 iterations. Note that for a given network size distance <*Dis*> tends to grow, approximately with the square root of the time elapsed since turning off the inputs (not shown), so those shown are not to be taken as the distances to any final, steady state position of the bump. Matching the trend of the spatial resolution, the average of the displacement drops to a value around 5 cm (one grid unit) for the largest network, but only for pre-wired connectivity with λ = 1. With λ = 2 < *Dis* > is much larger, and in the case of the self-organized connectivity resulting from the simulated Hebbian process, the mean displacement is larger still, and not abated below about 16 cm (≈3 grid units), even for relatively large networks (the largest size was not used, because of the CPU time required). One can use in this case as guides to the eyes the logarithmic trends:

(13) $$<Dis>=6.2-1.3\log_{10}\left(\frac{N_{CAs}}{\lambda^{2}}\right)$$

where one should note that the tuning width of the connectivity appears squared, λ^2^, and still in order to fit the data for the self-organized network one has to use the larger effective value λ = 5.0. This leads to much larger extrapolated network sizes, beyond which global metric distortion vanishes, that is <*Dis*>~ 0. One finds *N*_*CA*3_ ~58,800 for the pre-wired network with finely tuned connectivity (λ = 1); *N*_CA3_~235,000 with coarser connectivity (λ = 2); and *N*_CA3_~1,470,000 with self-organized connectivity. The final positions in the self-organized network are considerably more displaced than those in the networks pre-wired with either λ value, and their mean displacement does not seem to decrease much with size (Figure 2F), not rapidly enough, at least, to become irrelevant to a real-life sized rodent CA3 network of a few hundred thousand units. This suggests therefore that, with Hebbian learning, the recurrent network self-organizes in a virtual space of its own, which maps into real space in a much more indirect, less accurate way than in the case of a pre-wired network, even though the mapping can be similarly precise. This global distortion is illustrated in Figure 2C, which shows that to nearby locations in real space correspond somewhat more distant bumps on the attractor, but to distant positions in space correspond much closer bumps, on average, on the attractor. The effect stems from the toroidal geometry, but it indicates that the global distortion is alleviated very slowly, when increasing the size of the network. Further, these results suggest that attractor drift may be an unavoidable feature of self-organized attractors.

### Recurrent connections can preserve part of the input information

**Figure 3:**
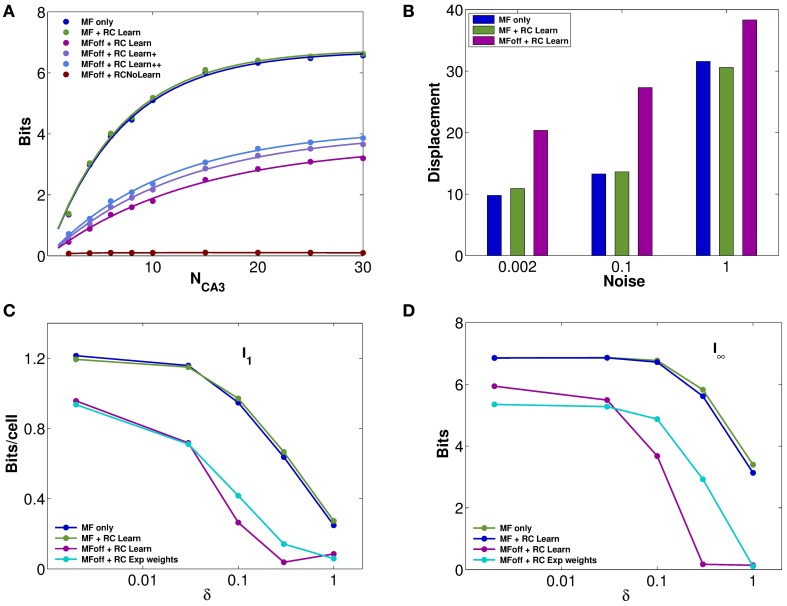
**Recurrent collaterals contribute little to the code, but sustain it, in part**. **(A)** Information plotted versus the number of CA3 units in the sample. Fast noise δ = 0.1. Blue curves refer to the DG-CA3 system without recurrent collaterals; adding the recurrent collaterals leads to the light green curves, and to the purple-light blue curves when mossy fiber inputs are turned off. The three purple to light blue curves refer to networks with DG inputs turned off, and recurrent connections self-organized with progressively more intense training, from bottom to top: simulations with 10,000 learning time steps and γ = 0.0001; with 10,000 training steps but γ = 0.002; and with γ = 0.002 but 20 × 10,000 training steps. The dark green curve (nearly flat) shows the spatial information present in the system when DG inputs are off, in the absence of any learning. **(B)** The bars show the mean displacement, as in Figure 2F, between the initial position and the final position for three values of fast noise, δ = 0.002, δ = 0.1, δ = 1. Averages over 400,000 trials, started randomly in one of the 400 possible initial positions, while decoding samples of 10 CA3 units. The limited sample used in decoding results in a meaningful mean displacement also when the network is still driven by DG inputs. (**C** and **D**) Information plotted versus the fast noise δ values, on a semi-logarithmic scale, as measured by the slope parameter of the information curve *I*_1_ **(C)** and by the saturation level *I*_8_ (**D**; see text). Blue, light green, and purple curves as above. Cyan curves correspond also to DG inputs off, but for a network with standard pre-wired exponential weights.

The impression, conveyed by Figures 2D–F, of similarly precise spatial attractor codes expressed by networks with exponential weights and with weights shaped by learning, is confirmed by information measures. They are extracted from simulations, as described in the Methods (and see Cerasti and Treves, 2010), by decoding sub-samples of different sizes of CA3 units, randomly picked from the network, resulting in trends like those shown in Figure 3A. Those trends are fitted as detailed in Equation (9), yielding estimates of the average information *I*_1_ conveyed by one unit, and of the information *I*_8_ conveyed by the entire population. These estimates confirm that pre-wired networks do not encode a more informative map than self-organized ones, at least when fast noise (fast in the sense that it changes from one theta cycle to the next) is effectively absent (i.e., of the order of δ = 0.002, the value used in Figures 1, 2). Figures 3C,D shows, in fact, that the mutual information encoded by an exponential pre-wired connectivity, with inputs turned off (cyan lines) is similar the one encoded by a network with Hebbian weights (purple lines). It turns out, however, to be slightly more resistant to noise, so for larger noise values the pre-wired network is somewhat more informative (compare the cyan line with the purple line in Figures 3C,D.

A discrepancy then arises between what we might call the *precision* and the *accuracy* of self-organized spatial codes. The information results extend to a wider noise range the indication obtained with the resolution and clustering measures of Figures 2D,F, pointing at the reasonably precise spatial codes that can emerge from self-organizing weights. Their precision is comparable to that of the ones artificially wired with exact exponential connectivity. In contrast, the analysis of the distance between initial and final positions, i.e., the mean displacement in Figure 2F, indicates that self-organized codes are considerably less accurate, in mapping real space, than the pre-wired ones. In other words, distinct spatial positions are resolved to a similar degree for both codes, but while exponential connectivity minimizes drift, thus preserving distances, the self-organized one does not. The discrepancy can be conceptualized by stating that the attractor “manifold” established by Hebbian learning has a metric of its own, different from the one of real space, and much closer in nature, instead, to the rowdy metric established by DG inputs. Figure 3B in fact shows that our measure of displacement, if computed by decoding small ensembles of only 10 units, yields substantial values already for the spatial code produced by DG inputs alone. Adding reverberation along recurrent connections does not alter <*Dis*> significantly. Once DG inputs are turned off, while a finely tuned prewired connectivity minimizes further drift by regularizing the attractor surface (not shown, but see Figure 2F), the self-organized connectivity adds substantial drift of its own (the higher purple bars in Figure 3, bottom right). An accurate metric map, however, is not necessary to maintain spatial knowledge in the network, with the result that the spatial information stored by the self-organized weights is not necessarily lower than with precisely wired weights. Such insight into the properties of these representations can be substantiated by applying information measures.

In terms of these measures, Figure 3 further quantifies, in a network with self-organized recurrent connectivity, how different connections contribute to the amount of spatial information present in limited samples of CA3 units. Three different conditions can be contrasted. In the first, MF inputs are active, while RC activity enters only as random noise, as in Cerasti and Treves (2010), see Table 1; in the second, recurrent connections are added, with weights resulting from the learning process, and MF inputs are kept active; in the third condition, information is extracted from the CA3 population in the absence of DG inputs, from the self-organized attractor.

RC activity seems not to add information to the CA3 representation, when the input coming from the MFs is fully available, as shown in Figures 3A–D (cp. blue and green data, for the self-organized network). The same holds for the pre-wired network (not shown). One has to take into account the relative strength of the two inputs arriving to a CA3 cell: in fact, in the simulations shown here, the MF weights are much stronger than RC ones, with a total mean strength 5 times larger (averaged over the whole environment). However, a similar result holds even when the strength of RC weights is increased as much as to equal total mean MF strength (not shown). When the input is turned off, the scenario changes and RC activity is crucial. Recurrent connections are, in fact, able to convey information about the stored spatial representation, as it is clear from Figures 3C,D, provided the noise is not too high.

**Figure 4:**
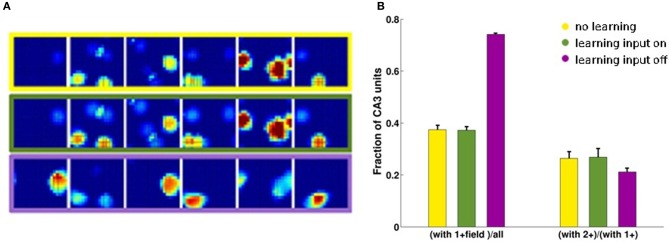
**CA3 place fields are refined by learning**. Examples of CA3 firing maps in the DG-CA3 model network with MF and RC connections. **(A)** The top row shows CA3 place fields with no Hebbian learning; the middle row shows the same fields after learning; and the bottom row shows them after mossy fiber inputs are turned off. **(B)** Fraction of CA3 units with at least a place field (3-bars group on the left) and fraction of those with multiple fields among those with at least one (3-bars group on the right). Same color coding as in the frames of the **(A)**.

Considering the effect of learning strength, for a fixed level of noise, Figure 3A shows the corresponding information curves plotted versus CA3 sample size, in the case of δ = 0.1. One can see the modest increase in the information due to a much higher learning rate, γ = 0.002, and to a much higher number of time steps, 20 × 10,000, in the model learning process. This indicates that protracted or intense learning (in terms of training time or learning rate) offers diminishing returns: most of the information structuring the attractor is acquired rapidly, even with minimal learning rates. The effect of the learning process on CA3 place fields can be seen in Figure 4A, where the firing maps in absence of MF inputs can be contrasted with the ones driven by them, with δ = 0.002. When MF inputs are turned off, RC activity generates several new fields or amplifies existing ones, and suppresses others already present, usually the weaker ones. The fraction of CA3 units with at least one place field increases significantly, whereas the proportion of these with 2 or more fields does not change (Figure 4B). This effect could be tested experimentally in the real system.

### Multiple charts leave residual spatial information

**Figure 5:**
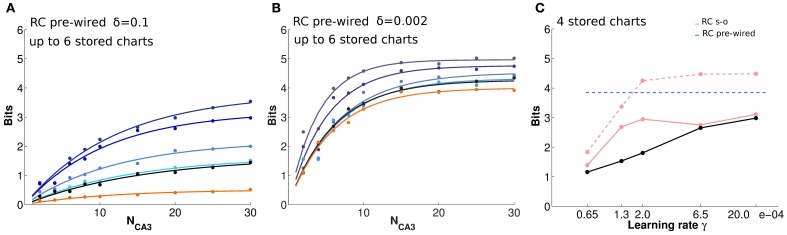
**The storage capacity for multiple charts is limited, but substantial residual information remains**. **(A** and **B)** Information plotted versus the number of CA3 units in the sample, for a substantial noise level, δ = 0.1 **(A)**, and for lower noise, δ = 0.002 **(B)**. The two panels show the average amount of information retained through pre-wired recurrent collateral weights, in absence of the input, about position in one environment, after the storage of 1, 2, 4, and 6 charts, indicated by curves from purple to light blue, i.e., from top to bottom. The black curves indicate the amount of information retrieved through recurrent collaterals about a chart that had never been stored, when 6 other charts were learned, the “residual information.” If no learning sessions had occurred, however, the curve would be flat at the bottom (not shown). The orange curve is the information retained after reshuffling synaptic weights, as explained in the text. Dots correspond to information values obtained from simulations, while the curves results from fits to the data points, as detailed in Methods. **(C)** Position information retrieved from the last (dashed pink) and first (pink) of 4 charts stored in a network with Hebbian learning, from 10 CA3 units, as a function of the learning rate. The dashed blue line is the reference level for 4 pre-wired charts. The black segments connect residual information datapoints. A full cue was provided to initiate retrieval. Limited plasticity (a small learning rate) is sufficient for effective storage of multiple chats, while increasing the rate, in this case beyond γ = 0.0002, adds only to the residual information.

Having confirmed the ability of the RC network to retain spatial information about a given environment, we study how such information is affected by the storage of multiple “charts,” i.e., representations of different environments. We allow the network, which in this case is comprised of 500 CA3 units, to store a different number of charts, each for an environment explored by the virtual rat for 3000 time steps. First, we consider the control case of exponential pre-wired efficacies (Figures 5A,B). In this case, all charts are encoded with equal strength. Figure 5A shows that a network of such limited size is overburdened already by the storage (i.e., the pre-wiring) of two charts: the mutual information that can be extracted about each from a given cell sample is below what can be extracted if only one chart is stored on the weights. There is a further decrease with the storage of four charts and, with six, the information in each of them is not more than what can be extracted about a chart that has *never* been stored. There is indeed significant information present in the network about positions in an environment that was not experienced in the simulations, and the amount becomes considerable when the noise level is low (Figure 5B). This apparently counterintuitive finding can be understood considering that information is not sensitive to the logic of the mapping between actual position and position decoded from population activity, but solely to the precision of such mapping; it turns out, from our measures, that a precise mapping can be “inherited” from other charts.

This “residual” information is not due to specific encoding on the weights but to the disorder (or inhomogeneity) created in the network by cumulative encoding of other information of the same spatial nature. In fact, the information available when weights are set to their initial uniform values and no storage occurs at all is essentially at zero (not shown). Is any inhomogeneity in the weights sufficient to produce residual information, or is the weight distribution resulting from the storage of a spatial representation in any way special? To address this question, we reshuffled the weights produced by the storage of 6 charts, and again measured the amount of spatial information retrieved, either about that chart or about another, non-stored chart. The result is the same, and the corresponding curve (orange in Figures 5A,B) is below the amount of residual information, though for low values of noise (central panel) considerably above the vanishing amount present with uniform weights. This indicates that a component of the residual information is merely due to the roughness of activity propagation in a recurrent network with generically inhomogeneous synaptic weights, while another component is due to the special spatial character of the inhomogeneity, due in turn to the storage of other charts, and not just to a sort of white noise. The difference between the two components probably lies in the variability of the distribution of weights presynaptic to any given unit, a point that requires further analysis.

These observations apply also to the case of self-organized representations. In this case, we run several simulations in which the network stores (self-organizes) representations of four different environments one after the other, and the degree of encoding is tested at the end for all charts, through decoding. The same length of exploration is used, but with different learning rates (Figure 5C). Obviously with such a procedure, the first chart to be stored is partially overwritten by the storage of successive charts; so that the amount of information about it is well below what can be retrieved about the last chart to be stored, especially with a high learning rate. For both, the encoding does not become stronger by using a learning rate above the intermediate value we used, γ = 0.0002, and information about the first and last chart “stabilizes” at amounts below and above the average across the four charts, which is roughly what can be retrieved from a pre-wired network (the horizontal line in Figure 5C; in the pre-wired case, obviously all charts are stored with statistically equal strength). This is the spatial equivalent of one-shot learning in episodic memory: virtually all there is to learn can be acquired with small modifications of the connection weights, and there is almost no return from more intense learning.

Interestingly, information about an environment that was never experienced, what we have called *residual* information, continues to grow a bit further with an increase in the learning rate, and approaches the amount relative to the first chart encoded for γ = 0.00065. From this rate of learning onward, the storage capacity of the network, with our parameters, is below 4 charts, and what is present about the first chart, which has been all but “forgotten,” is just the residual information, which is however very substantial (when noise is low, δ = 0.002).

This finding is broadly consistent with the notion, put forward over several years by Bruce McNaughton, that spatial charts are somehow prewired in the hippocampal system, and that spatial experience merely serves to associate locations in a pre-wired chart with configurations of sensory cues available in that location (Colgin et al., 2010). In McNaughton's view, the pre-existing chart is expressed by the path-integration system, which may well operate upstream of the hippocampus, e.g., in medial EC. Our model is not in conflict with this view, as it makes no distinction, in fact, between inputs to CA3 that relay path integration information vs. sensory information, e.g., between those on the medial vs. lateral perforant path that relay inputs from the medial and lateral EC to both DG and CA3. There is instead the distinction between (DG) inputs and recurrent connections. Still, the experimental evidence for the existence of prewired charts can be interpreted as also consistent with the considerable spatial information that, in a novel environment, may be available in recurrent weights modified by the charts of other environments—what we see in the model and have called residual information.

### Context information is also retrieved and partially maintained in the attractor

As we have discussed elsewhere (Stella et al., 2012), the specific contribution of the CA3 network can be argued to be in the formation of memories for specific spatio-temporal contexts, more than in retaining spatial information about a single context. So far we have quantified the amount of spatial information in one chart, either the only one or one among several stored concurrently, but one can also ask how much information the network can produce about which chart best matches the current environment, irrespective of exact position within it. Can CA3 tell a real rat not just where it is in one context, but also which context it is, among several competing possibilities? Can it retain this information in memory, and reactivate it from partial sensory cues? The question is made particularly relevant by the observation that the remarkable spatial code observed in the medial EC, expressed by grid cells, while providing exquisitely fine information about the position of the animal (Burak and Fiete, 2009) appears unable to discriminate between contexts (Fyhn et al., 2007).

We have addressed this question, again, by simulating the storage of 4 distinct charts, obtained by globally remapping the DG activity the drives the establishment of CA3 charts in the model, and again assessing both the representations activated by the inputs and those remaining active when the inputs are turned off. Self-organized charts can again be compared with those manually pre-wired.

**Figure 6:**
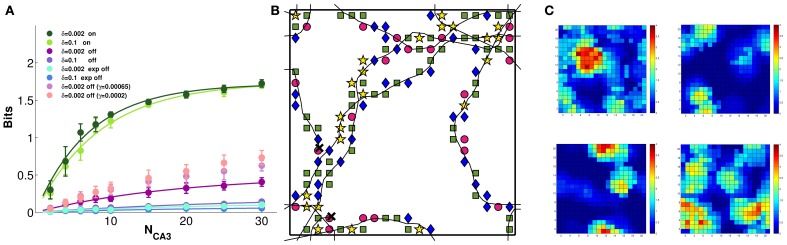
**Some context discrimination remains in memory, if self-organization is limited**. The amount of information about which of 4 distinct environments is reflected in the DG inputs is extracted from subsamples of CA3 model units. **(A)** Decoding the activity driven by the inputs allows for good discrimination (the green curves, which are fitted to the data points as before), irrespective of noise. Turning the inputs off greatly reduces the context information retained in all cases, but more information remains when the network is allowed to self-organize, and noise is very low (purple curve). In contrast, pre-wired connectivity appears not to be suitable for context discrimination (light and dark blue curves). Importantly, context information decreases with more plasticity (pink to purple datapoints, top to bottom), indicating progressive attractor collapse. **(B)** A sample 300-time-step trajectory, with the environment decoded at each position, from a random sample of 10 units, shown by a different symbol (the green square is the correct environment). Some of the locations are superimposed on the 10 × 10 grid used for decoding. **(C)** The cumulated activity of the 10 units in each of the 4 environments, plotted in pseudo-colors. The correct environment is top left. Typically 3–4 of the units have a field in each environment, occasionally a double field. Hence overall activity remains sparse.

Figure 6A shows that the model CA3 network can easily discriminate between 4 different contexts, even based on the activity of a limited sample of units, when DG inputs are still on. The discriminative capacity reaches close to the maximal value of 2 bits, irrespective of the noise level. When the inputs are turned off, the amount of retained context information becomes very sensitive to the noise level, and is minimal for relatively higher levels of noise. The interesting observation is that, for a given noise level, context information grows with the learning rate, which is intuitive, but only up to γ = 0.0002 (not shown); beyond that learning rate, less information can be retained the higher the learning rate. The three sets of mauve datapoints in Figure 6A correspond to the three larger learning rates of Figure 5C: γ = 0.0002, 0.00065, and 0.002 (see the legend of Figure 6). Thus multiplying the learning rate by 10, which brought no benefit to spatial information (Figure 5C), actually causes a loss of context information (Figure 6A). This is due to different charts collapsing onto each other, to some degree, so that individual fragments of one “stick” to those of another, and context discrimination is impaired. The model therefore suggests that self-organization cannot be “pushed” beyond its own limits: rather than smoothing out the granularity of the charts (which is there even when they are pre-wired) or stretching out their global metric (which is more distorted in self-organized than in pre-wired charts) stronger self-organizations leads to gluing the charts with each other, like overcooked fettuccine.

Figure 6B shows which context is decoded from the activity of a sample of 10 units at successive positions along 300 time steps of a sample trajectory (when DG inputs are turned off): although the correct context is decoded more frequently than the other 3, the relative proportions are comparable, which makes the available context information low. Figure 6C shows one reason for such poor decoding performance: the overall activity of those 10 units, even when summed together, is sparse, in each of the 4 contexts. At many positions, there are no cells in the sample active, in a particular context. The other reason, of course, is related to the drift analysed previously. Interestingly, a pre-wired network appears to retain much less context information than a self-organized one, even when noise is low. This stands in contrast to the more accurate representation of spatial position expressed by the pre-wired network, and may be due to the way the pre-wiring procedure prevents the spontaneous development of context specific features of the spatial code.

## Discussion

The idealized notion of a continuous 2D attractor, a “chart,” has been an important guide for theories of how the hippocampus operates in spatial memory (Samsonovich and McNaughton, 1997; Stringer and Rolls, 2002; Stringer et al., 2002; McNaughton et al., 2006). Simulating the formation of such charts in CA3, we find that what emerges is considerably distant from the idealized concept. Rather than “flaws” in the representation expressed by the network, the discrepancies from the ideal 2D spatial attractor point, in our view, at what CA3 is really meant to do: not merely recode spatial coordinates, but embed (some) spatial information into new memory representations.

We have compared a model network with self-organized connectivity with the control case of a network pre-wired with a connectivity dependent on the exact distance among the place fields produced by a randomizing dentate input, and focused on three distinct aspects to characterize CA3 attractors: their spatial precision; their accuracy; their informative content relative to one or more environments.

Networks of finite size cannot express a truly smooth continuous attractor manifold, but rather a quasi-continuous collection of discrete attractor points, characterized, in statistical physics parlance, as distinct valleys of the free-energy landscape. Left to their own recurrent dynamics, networks cannot settle into a pattern of activity that represents any possible position in space, but only a discrete, “granular” subset, leading to a decrease in spatial resolution; when driven by afferent inputs, the continuity is retrieved (Kali and Dayan, 2000). This “roughness of the free-energy,” or imprecision in representing local continuity, had first been noted by Tsodyks and Sejnowski (1995), in a pre-wired one-dimensional model of a spatial attractor. Like Brunel and Trullier (1998), we find that also two-dimensional attractors “suffer” from these finite size effects; however, in both cases of pre-wired and self-organized networks, the roughness is seen to essentially disappear when reaching the size of a real rodent hippocampus (Figures 2D–F).

Moreover, the self-organization process turns out to be quite rapid (as in Nakazawa et al., 2003), effectively the spatial equivalent to one-shot learning in episodic memory: Figure 3A shows that more intense training brings very limited added value.

A pre-wired network with place fields regularly arranged on a grid would be much smoother, just like the entorhinal grid network, which is thought to express a single spatial representation (Fyhn et al., 2007) and to gradually smoothen it during development (McNaughton et al., 2006; Kropff and Treves, 2008). Such regular arrangement is however an artificial condition, particularly in the CA3 region, where providing strong, sparse and necessarily random inputs appears to be the raison d'être of the DG. Kali and Dayan (2000) consider a pre-wired and a self-organizing recurrent CA3 network like ours, and they also come to the conclusion that self-organized attractor manifolds can be similarly smooth as those determined by suitable pre-wired connectivity.

A second, conceptually distinct discrepancy from the idealized notion of a true representation of space is in the global geometry of the attractor manifold. This can be quantified indirectly by measuring the mean drift, i.e., the displacement, of the position decoded by the activity in the network from the position provided as input, which can be referred to as *global distortion* or *metric distortion*. In this respect, a self-organized attractor is considerably more distorted than a pre-wired one. Kali and Dayan (2000) discuss how the global distortion resulting from grossly uneven sampling of the environment can be effectively removed by modulating synaptic changes by a familiarity factor, which essentially reduces learning in areas that have been oversampled by the real or the virtual rat—typically, those at the center of the arena. The point they make is susceptible of experimental validation. In our simulation paradigm, however, the periodic boundary conditions do not give rise to gross oversampling of particular areas, so the global distortion in the attractor representation does not arise from that, but from random fluctuations. We find that the effect of such random fluctuations is not abated, as it might have been expected, in large self-organizing networks, which remain much more globally distorted than pre-wired networks in their spatial representations. Real-life networks may then suffer from a degree of metric distortion similar to the one observed here in a reduced model, although it is possible that the distortion be attenuated by additional mechanisms not considered here, such as short-term gain enhancement or synaptic facilitation (Roudi and Treves, 2008; Itskov et al., 2011). It could well be, as recently hypothesized by Romani and Tsodyks (2010), that the tendency to drift is expressed also as a propensity to replay and even preplay spatial trajectories, as observed in many experiments (e.g., Wilson and McNaughton, 1994; Foster and Wilson, 2006; Diba and Buzsáki, 2007; Karlsson and Frank, 2009; Dragoi and Tonegawa, 2010; Gupta et al., 2010). Replay would not reflect purposeful “thoughts” but rather uncontrolled drift along a randomly self-organized chart, whether relative to the current environment or residual from a previous environment. The difficulty with which (rapidly) self-organized representations come to reproduce global geometry contrasts with the beautiful, long-distance encoding of geometry by entorhinal grid cells (Hafting et al., 2005) which, again, presumably requires long developmental times to be established (Kropff and Treves, 2008). Once more, it appears how the CA3 network is not ideally suited to compute spatial relations *per se*, but rather to encode in memory spatial relations already computed upstream.

The spatial code expressed in the attractor is made inaccurate anyway by sampling only a small fragment of the entire population vector, as is the case with experimental recordings (Wilson and McNaughton, 1993). In fact, Figure 3B shows that populations of limited size are already quite inaccurate in the presence of the inputs, so that turning off the inputs, and letting activity to be sustained by the attractor, brings about only an additional degradation (e.g., doubling the mean displacement). In the light of our context information analysis, inaccurate spatial codes appear as a small price to pay in order to form arbitrary episodic memories with spatial content. In our simplified model, non-spatial aspects of such memories are not included explicitly, but still their episodic character emerges indirectly from the difference between the full and simplified localization matrices, the “dark information” discussed by Cerasti and Treves (2010).

The third dimension of discrepancy from the idealized notion requires, to be appreciated, information measures. Compared to the control, pre-wired network, Figures 3C,D show that a single environment can be encoded, in information terms, just as well with a self-organizing chart. Ideally, however, it should be possible to store multiple independent spatial attractors in the same recurrent network, to represent distinct environments with complete remapping from one to the other (Leutgeb et al., 2005), up to a capacity limit which has been studied analytically by Battaglia and Treves (1998). In our study, with the parameters we use and with the extra disorder of the irregular arrangement of the DG-induced fields, it appears that the weight modifications that encode a new chart, comprised of the cumulative granular “memory” of many different positions in the environment, are sufficient to almost wash out the information about a different environment learned in a previous session, even the one before. We say “almost,” because of a paradoxical counter-effect. We find, in fact, that the network still encodes residual information about charts it has effectively “forgotten,” and even about charts it has never stored in memory. Such “residual information” is limited when the noise, i.e., the temporal variability, is high (Figure 5A), but still it has to be taken into account when using decoding procedures also with real data. This effect can be explained by considering that, unlike temporal variability (noise), fixed or “quenched” spatial variability (disorder) causes reproducibility, which counter-intuitively confers information also to a random neuronal code. A component of this information even survives the reshuffling of all the synaptic weights.

Our overall conclusion is that, as a memory network, CA3 is clumsy at handling spatial information. If this runs against the intuition accrued from experiments, it may be because many of those experiments do not really probe memory function: they assess CA3 activity largely with “the inputs on.” It is unclear to what extent DG inputs are reduced, during free exploration and foraging, even at their lowest theta phase. Nevertheless, it is important to analyze attractor dynamics as a function of theta phase, as in the study revealing brief, theta-paced “flickering” memory dynamics in CA3 (Jezek et al., 2011). Even more important is to look at the information putatively retrieved from CA3 during sharp wave ripples (SWR). SWR events are thought to originate in CA3, presumably with no need for the dentate to trigger them with spatial information. Their statistical character is very different from what is observed during theta-modulated states, and they may have to do more with long-term memory formation elsewhere than with the active short-term retention of spatial information in hippocampal networks (Hoffman et al., 2007). They may be suited, therefore, to characterize attractors formed in CA3 during theta-modulated spatial exploration (Nakazawa et al., 2003).

### Conflict of interest statement

The authors declare that the research was conducted in the absence of any commercial or financial relationships that could be construed as a potential conflict of interest.
